# Gadolinium Clearance in the First 5 Weeks After Repeated Intravenous Administration of Gadoteridol, Gadoterate Meglumine, and Gadobutrol to rats

**DOI:** 10.1002/jmri.27693

**Published:** 2021-05-11

**Authors:** Simona Bussi, Alessandra Coppo, Roberta Bonafè, Silvia Rossi, Sonia Colombo Serra, Laure Penard, Miles A. Kirchin, Federico Maisano, Fabio Tedoldi

**Affiliations:** ^1^ Bracco Imaging SpA, Bracco Research Centre Colleretto Giacosa TO Italy; ^2^ Charles River Saint Germain‐Nuelles Lyon France; ^3^ Bracco Imaging SpA, Global Medical & Regulatory Affairs Milan Italy

**Keywords:** gadolinium retention, gadoteridol, gadoterate, gadobutrol, rats, GBCA

## Abstract

**Background:**

Studies of gadolinium (Gd) clearance from animals in the first weeks after administration of gadolinium‐based contrast agents (GBCAs) have previously looked at solitary timepoints only. However, this does not give information on differences between GBCAs and between organs in terms of Gd elimination kinetics.

**Purpose:**

To compare Gd levels in rat cerebellum, cerebrum, skin, and blood at 1, 2, 3, and 5 weeks after repeated administration of macrocyclic GBCAs.

**Study Type:**

Prospective.

**Animal Model:**

One hundred eighty male Sprague–Dawley rats randomized to three groups (*n* = 60/group), received intravenous administrations of gadoteridol, gadoterate meglumine, or gadobutrol (0.6 mmol/kg for each) four times/week for 5 consecutive weeks. Rats were sacrificed after washout periods of 1, 2, 3, or 5 weeks.

**Field Strength/Sequence:**

Not applicable.

**Assessment:**

Cerebellum, cerebrum, skin, and blood were harvested for Gd determination by inductively coupled plasma‐mass spectrometry (15 animals/group/all timepoints).

**Statistical Tests:**

Anova and Dunnett's test (data with homogeneous variances and normal distribution). Kruskal–Wallis and Wilcoxon's rank sum tests (data showing nonhomogeneous variances or a non‐normal distribution, significance levels: *P* < 0.05, *P* < 0.01, and *P* < 0.001).

**Results:**

Gd levels in cerebellum, cerebrum, and skin were significantly lower after gadoteridol than after gadoterate and gadobutrol at all timepoints. Mean cerebellum Gd concentrations after gadoteridol, gadoterate, and gadobutrol decreased from 0.693, 0.878, and 1.011 nmol Gd/g at 1 week to 0.144, 0.282, and 0.297 nmol Gd/g at 5 weeks after injection. Similar findings were noted for cerebrum and skin. Conversely, significantly higher Gd levels were noted in blood after gadoteridol compared to gadobutrol at 1, 2, and 3 weeks and compared to gadoterate at all timepoints.

**Data Conclusion:**

Gadoteridol is eliminated more rapidly from rat cerebellum, cerebrum, and skin compared to gadoterate and gadobutrol in the first 5 weeks after administration, resulting in lower levels of retained Gd in these tissues.

**Evidence Level:**

1

**Technical Efficacy:**

Stage 5

Gadolinium‐based contrast agents (GBCAs) are used in approximately one‐third of all MRI examinations worldwide.^1^ It is estimated that roughly 50 million doses are given annually, and that more than 500 million doses have been administered since their introduction in 1988.^1^ Nevertheless, the safety of GBCAs has come under scrutiny in recent years, initially due to a causative association with the development of nephrogenic systemic fibrosis (NSF) in patients with severe renal insufficiency^1^ and more recently due to the observation of signal hyperintensity in the dentate nucleus (DN) and globus pallidus (GP) on unenhanced T1‐weighted images of patients with prior GBCA exposure.^2^ Subsequently, findings of retained gadolinium (Gd) in brain tissue samples from decedents undergoing autopsy^3^ have further elevated concerns over their use. Studies to date have shown that most cases of NSF and brain T1 hyperintensity occur after exposure to linear GBCAs rather than macrocyclic GBCAs and that there are marked differences between the simple linear GBCAs (i.e. gadodiamide and gadopentetate dimeglumine) and the substituted linear GBCAs (gadobenate dimeglumine and gadoxetate disodium) with no cases of NSF reported for the latter agents.^1^

Unfortunately, because determination of Gd levels in living humans is impractical, most studies on Gd retention have been performed in animals (primarily rats). These studies have largely confirmed differences between linear and macrocyclic GBCAs^4^, ^5^, ^6^, ^7^, ^8^, ^9^, ^10^ and between simple and substituted linear GBCAs^11^ in terms of the amount of Gd retained after single or daily cumulative exposures. Interestingly, however, animal studies have also revealed differences among the macrocyclic GBCAs in terms of the amount of Gd retained, particularly in the first days and weeks after exposure, with lower levels of Gd retained in rat brain and body tissues after cumulative exposure to gadoteridol than after cumulative exposure to identical doses of gadoterate meglumine and gadobutrol, suggesting more rapid clearance of gadoteridol in the first weeks after injection.^11^, ^12^, ^13^, ^14^ A previous study by Jost et al^14^ looked at the long‐term elimination of Gd after cumulative GBCA exposure but did not begin determination of Gd levels in brain tissues until 5 weeks after the final GBCA administration. However, the initial postinjection period may be critical in reducing the amount of Gd available for longer‐term retention and thus faster GBCA elimination during this period may be potentially important, especially when repeated administrations of GBCAs are planned.

To date, studies that have compared Gd retention in the first weeks after GBCA administration have looked at solitary timepoints, allowing only inferential conclusions to be drawn regarding the kinetics of Gd elimination. Our aim was to more closely investigate and compare the elimination profiles of the macrocyclic GBCAs in rats in the first weeks after cumulative administration.

## Materials and Methods

The study was performed at Charles River Saint Germain‐Nuelles (France), American Association for Accreditation of Laboratory Animal Care (AAALAC) accredited, according to site‐specific procedures established by the relevant Quality Assurance Unit. Procedures were conducted according to national and international regulations for animal welfare (Decree 2013‐118 relating to the protection of animals used in scientific experiments described in the *Journal Officiel de la République Française* on February 01, 2013; Directive 2010/63/EU).

### Animal Model and GBCA Administration Protocol

One‐hundred eighty male Sprague Dawley OFA rats (Charles River Laboratories, France) aged 6 weeks and weighing between 141 g and 244 g at the start of treatment were utilized. Three GBCAs were compared: gadoteridol (ProHance; Bracco Imaging SpA, Milan, Italy; batch no. V19732, expiry: June 2022), gadoterate meglumine (Dotarem; Guerbet LLC, Villepinte, France; batches no. 18GD008A01 and 20GD001A02, expiry: February 2021 and December 2022, respectively) and gadobutrol (Gadovist; Bayer, Leverkusen, Germany; batch no. KT05367, expiry: August 2022).

Animals were housed under controlled conditions at a temperature of 22°C ± 3°C, humidity of ≥35% and 12‐h light/dark cycles. Food pellets and filtered water from municipal services were provided ad libitum. After 7 days of acclimation, animals were randomly assigned to one of three exposure groups: group 1 (gadoteridol, 0.5 mol/L), group 2 (gadoterate meglumine, 0.5 mol/L), and group 3 (gadobutrol, 1 mol/L).

All rats received a daily intravenous injection of GBCA at a dose of 0.6 mmol/kg four times a week (four consecutive days, Tuesday to Friday), for five consecutive weeks (i.e. 20 administrations overall), for a total cumulative dose of 12 mmol/kg. This daily dose corresponds to a human clinical dose of 0.1 mmol/kg.^15^ GBCA administration was performed at room temperature into the lateral vein of the tail at 2 mL/min using a Harvard infusion pump (Holliston, MA).

**FIGURE 1:**
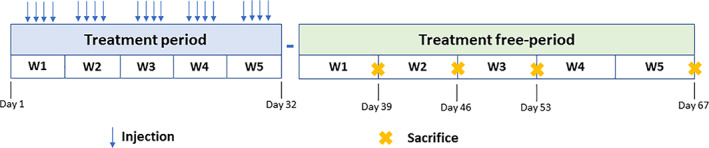
Experimental design. Rats were randomized to three exposure groups (*n* = 15 per group). Animals in each group received single daily intravenous injection of the respective GBCA at a dose of 0.6 mmol/kg bodyweight four times a week for 5 consecutive weeks. After the end of the 5‐week treatment period (day 32), 15 animals in each group were sacrificed at 1 (day 39), 2 (day 46), 3 (day 53) or 5 weeks (day 67) after the last injection and blood and tissues harvested for Gd determination by ICP‐MS.

Animals in each group were sacrificed after washout (treatment‐free) periods of 1, 2, 3 or 5 weeks after the end of the 5‐week treatment period (15 rats per group per timepoint: Fig. 1).

### Observations

During the treatment period, all animals were inspected before and after dosing for any clinical signs or reactions to treatment. During the treatment‐free period, all animals were inspected once daily and weighed weekly. A full clinical examination was performed pretest and then weekly during the treatment and treatment‐free periods.

### Pathology

Animals were sacrificed by carbon dioxide inhalation followed by exsanguination. Animals were euthanized by group to avoid cross‐contamination. After exsanguination, a complete macroscopic postmortem examination was performed which included evaluation of the carcass and musculoskeletal system; injection sites; all external surfaces and orifices; cranial cavity and external surfaces of the brain; and thoracic, abdominal, and pelvic cavities with their associated organs and tissues. Abnormal findings, if present, were recorded by the lead histopathologist (with 20 years of experience). Thereafter, each animal was dissected to obtain tissues (cerebellum, cerebrum, and skin) for the determination of gadolinium by inductively coupled plasma/mass spectrometry (ICP‐MS). Tissues were sampled from all animals, using clean or disposable materials (to avoid cross‐contamination), weighed, immediately placed on dry ice and then frozen at −80°C ± 10°C. A total of 720 blood/tissue samples were collected (180 animals; 3 tissue samples, and 1 blood sample per animal).

### Determination of Total Gd

All procedures were carried out at the Arcinova Test Site (Alnwick, Northumberland, UK). Brain tissue and skin samples were stored at −80°C prior to digestion. Following digestion but prior to analysis, samples were stored at −20°C. Blood samples were refrigerated at +2/+8°C. All blood and tissue samples were digested with 4 volumes of 70% (w/w) nitric acid overnight at room temperature. An aliquot (2.5 mL) of internal standard solution was combined with 50 μL of sample digest, capped, briefly vortex mixed and then rotary mixed for at least 30 minutes. The processed sample was analyzed by ICP‐MS on an Agilent 7700x or 7900 inductively coupled plasma mass spectrometer.

Blood and tissue Gd concentrations (nmol Gd/g) were calculated using digest Gd concentrations calculated in nmol/mL, corrected for sample dilution during digest preparation and converted from *per* mL of solution to *per* g of wet sample. The lower limit of quantitation (LLOQ) for Gd was 0.032 nmol Gd/g.

### Data Fit and Calculation of Elimination Half‐Lives

Elimination half‐lives (t_1/2_) were calculated as τ∙log 2, with τ being the best fitting parameter obtained by interpolation of data with a mono‐exponential equation:

$$y\left(t\right)=A\;e^{-\frac{t}{\tau }}$$

The same equation, with the best fitting parameters, was also applied to extrapolate the Gd concentration at time *t* = 1 day after dosing.

### Statistical Analysis

Gadolinium concentration was expressed as nmol Gd/g wet tissue. All statistical tests were conducted at the 5% significance level. All pairwise comparisons were conducted using two sided tests and were reported at the 1% and 5% levels, unless otherwise noted. Before formal analysis of ICP data, the Dixon test^16^, ^17^ was used to detect anomalous/outlier data points, using the “critical value for Q parameter table” from Rorabacher^18^ and a 95% confidence level. Levene's test^19^ was used to test the equality of variance across groups and Shapiro–Wilk's test^20^ to assess the normality of data distribution in each group. Data with homogeneous variance and normal distribution in all groups were analyzed using Anova followed by Dunnett's test.^21^ Data showing nonhomogeneous variance or a non‐normal distribution in at least one group were analyzed using the Kruskal–Wallis test followed by Wilcoxon's rank sum test^21^ (significance level up to 0.1%). All statistical analyses were performed at Charles River, using SAS software, version 8.2 (Cary).

Gd content extrapolated at *t* = 1 day and elimination half‐lives were compared pairwise by applying a two‐sample z‐test for comparing two values μ1 and μ2, with errors σ1 and σ2. Errors were estimated by the fitting procedure (for half‐lives) and through error propagation rules for Gd content at 1 day. The null hypothesis was that the two values are equal, while the alternative hypothesis was that the two values are different. The z‐value was calculated as:

$$z‐\text{value}=\left(\text{μ1}-\mu 2\right)/\text{sqrt}\left({\text{σ1}}^{2}+{\text{σ2}}^{2}\right)$$

and compared with critical values 1.96, 2.57, and 3.29, corresponding to a significance of 5%, 1%, and 0.1%, respectively. Statistical analyses were performed using Mathematica software, version 12.0 (Wolfram).

## Results

All animals successfully underwent all aspects of the study and were sacrificed as foreseen in the study protocol. No unexpected changes in bodyweight were noted and no adverse signs or symptoms were observed for any animal. No relevant gross pathological tissue changes related to GBCA administration were noted at sacrifice.

**FIGURE 2:**
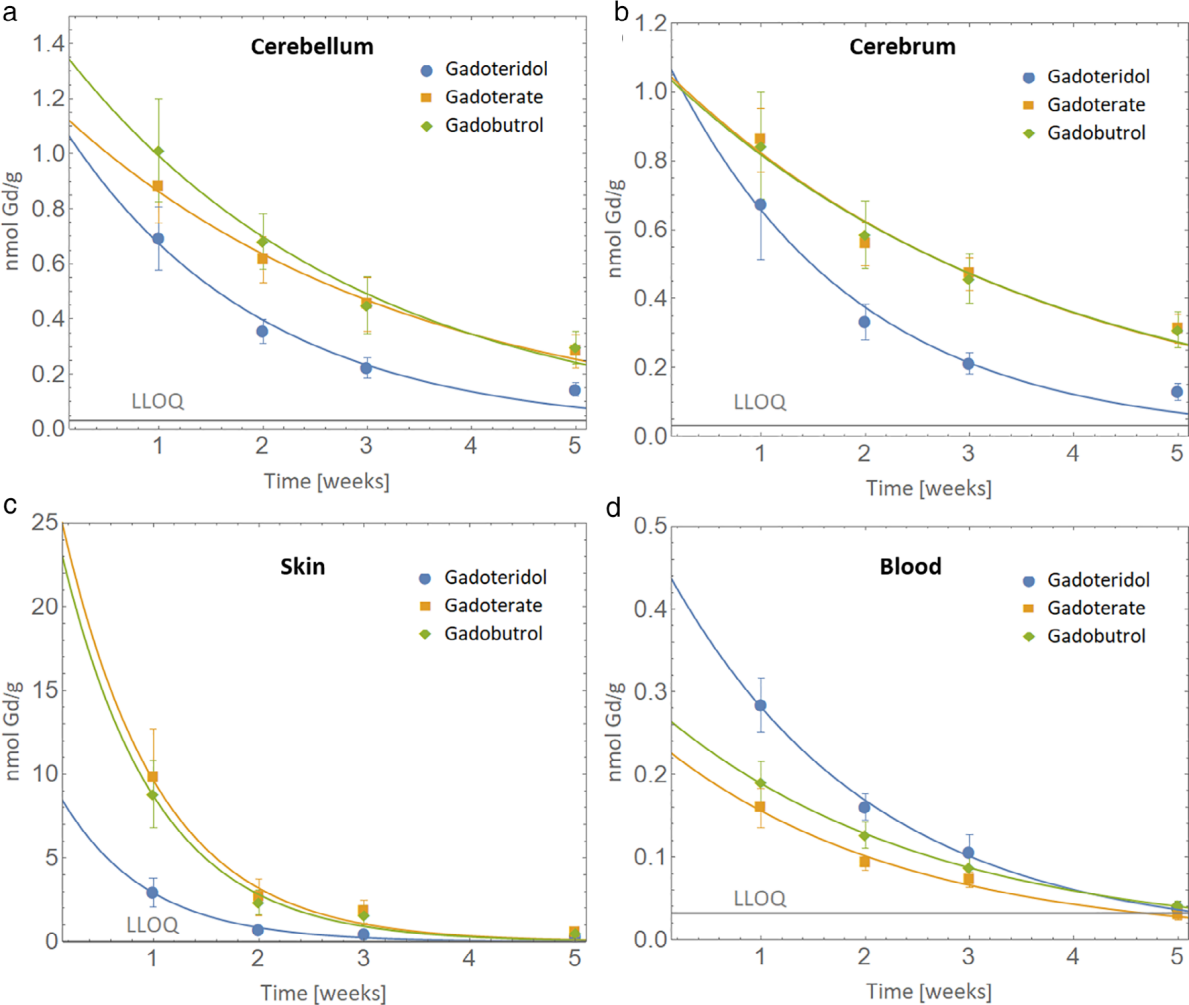
Gadolinium content in (a) cerebellum, (b) cerebrum, (c) skin and (d) blood: mean values ± SD. The error bars represent the standard deviation of measurements within the groups (*n* = 15, except for “Gadoteridol skin, 2 and 3‐week recovery,” “Gadoterate cerebellum, 2‐week recovery,” “Gadoterate cerebrum, 2 and 3‐week recovery,” and “Gadoterate skin, 5‐week recovery,” where *n* = 14). Solid lines represent the best fitting according to a mono‐exponential equation.

The mean (± Standard Deviation, SD) Gd contents across groups and tissue types are presented in Table 1 and Figure 2. Among the 720 samples, data points from six (0.008%) samples were considered outliers based on the Dixon test. No more than a single outlier was identified in each 15‐sample group of animals and organs. These data points were excluded from further elaboration.

**TABLE 1:** Gadolinium Content in the Cerebellum, Cerebrum, Skin, and Blood

	One‐week Recovery	Two‐weeks Recovery	Three‐weeks Recovery	Five‐weeks Recovery
Cerebellum (nmol Gd/g)				
Gadoteridol	0.693 ± 0.114	0.356 ± 0.043	0.223 ± 0.037	0.144 ± 0.025
*n*	15	15	15	15
Gadoterate	0.878 ± 0.130***	0.614 ± 0.083***	0.453 ± 0.097***	0.282 ± 0.061***
*n*	15	14	15	15
Gadobutrol	1.011 ± 0.187***	0.682 ± 0.100***	0.449 ± 0.104***	0.297 ± 0.059***
*n*	15	15	15	15
				
Cerebrum (nmol Gd/g)				
Gadoteridol	0.675 ± 0.163	0.332 ± 0.052	0.212 ± 0.031	0.129 ± 0.024
*n*	15	15	15	15
Gadoterate	0.860 ± 0.092**	0.558 ± 0.063***	0.471 ± 0.047***	0.311 ± 0.042***
*n*	15	14	14	15
Gadobutrol	0.843 ± 0.156**	0.585 ± 0.098***	0.457 ± 0.072***	0.309 ± 0.052***
*n*	15	15	15	15

Skin (nmol Gd/g)				
Gadoteridol	2.939 ± 0.854	0.694 ± 0.162	0.460 ± 0.079	0.305 ± 0.068
*n*	15	14	14	15
Gadoterate	9.744 ± 2.933***	2.651 ± 1.077***	1.791 ± 0.684***	0.525 ± 0.142***
*n*	15	15	15	14
Gadobutrol	8.799 ± 1.998***	2.328 ± 0.738***	1.582 ± 0.578***	0.518 ± 0.184***
*n*	15	15	15	15

Blood (nmol Gd/g)				
Gadoteridol	0.284 ± 0.033	0.160 ± 0.016	0.106 ± 0.021	0.039 ± 0.006^a^
*n*	15	15	15	15
Gadoterate	0.159 ± 0.024***	0.092 ± 0.008***	0.072 ± 0.009***	0.029 ± 0.005***^b^
*n*	15	15	15	15
Gadobutrol	0.190 ± 0.026***^,^°°	0.126 ± 0.016***^,^°°°	0.087 ± 0.017**^,^°°	0.042 ± 0.005°°°^c^
*n*	15	15	15	15

Values presented as mean ± Standard Deviation (SD).

The significance of differences between groups is shown as *P ≤ 0.05, **P ≤ 0.01, ***P ≤ 0.001 vs. group 1 and °*P* ≤ 0.05; °°*P* ≤ 0.01; °°°*P* ≤ 0.001 vs. group 2.

Data for blood below the lower limit of quantification (LLOQ) were extrapolated.

^a^ 3/15 value below the quantification limit.

^b^ 10/15 values below the quantification limit.

^c^ 1/15 value below the quantification limit.

Measurable amounts of Gd were detected in all tissue samples up to 5 weeks after the last injection. Extrapolated values were used for blood samples at the 5‐week timepoint, when the amount of Gd was below the LLOQ. Extrapolated values corresponded to at least three times the background, based on instrument response to a reagent blank sample. Significant differences in elimination kinetics were noted for the three GBCAs in all tissues and at all timepoints.

### Cerebellum and Cerebrum

Significantly lower levels of Gd were found with gadoteridol compared to gadoterate and gadobutrol in both cerebellum and cerebrum at all tested timepoints (Table 1). Conversely, no significant differences were noted between gadoterate and gadobutrol at any timepoint (cerebellum: *P* = 0.11, *P* = 0.08, *P* = 1.0, *P* = 0.56; cerebrum: *P* = 0.79, *P* = 0.63, *P* = 0.76, *P* = 0.77; at 1, 2, 3, and 5 weeks, respectively). The mean cerebellum Gd concentrations at 1 week after the last injection (i.e. after 1 week of GBCA washout) were 0.693 ± 0.114, 0.878 ± 0.130, and 1.011 ± 0.187 nmol Gd/g for gadoteridol, gadoterate, and gadobutrol, respectively. At week 5 after the last injection the Gd concentrations in the cerebellum had fallen to 0.144 ± 0.025, 0.282 ± 0.061, and 0.297 ± 0.059 nmol Gd/g, respectively. Corresponding values for the cerebrum were 0.675 ± 0.163, 0.860 ± 0.092, and 0.843 ± 0.156 nmol Gd/g for gadoteridol, gadoterate and gadobutrol, respectively, at week 1 and 0.129 ± 0.024, 0.311 ± 0.042, and 0.309 ± 0.052 nmol Gd/g, respectively, at week 5 after the last injection.

Notwithstanding the lowest levels of Gd in brain tissues at 1 week after the end of gadoteridol dosing, the levels of Gd determined after gadoteridol administration continued to decrease more quickly over the following weeks. At 5 weeks after gadoteridol administration, the Gd levels in the cerebellum and cerebrum decreased by a factor of about 5 compared to the levels found at week 1. In contrast, the corresponding Gd levels for gadoterate and gadobutrol decreased only by a factor of about 3, indicating a more rapid elimination rate for gadoteridol than for gadoterate and gadobutrol from the cerebellum and cerebrum during the initial 5 weeks after administration.

### Skin

As in the cerebellum and cerebrum, significantly lower levels of Gd were noted in the skin at all timepoints after gadoteridol administration than after administration of gadoterate or gadobutrol (Table 1), while no differences were noted between gadoterate and gadobutrol (*P* = 0.41, *P* = 0.51, *P* = 0.43, *P* = 0.65 at 1, 2, 3 and 5 weeks, respectively). After 1 week of washout, the Gd levels after gadoteridol administration were about three‐fold lower than those after gadoterate or gadobutrol administration. At 5 weeks after the end of dosing, Gd levels were about 1.7 times lower after gadoteridol administration than after gadoterate or gadobutrol administration.

### Blood

Significantly higher levels of Gd were found after gadoteridol administration at all timepoints up to 3 weeks after injection (Table 1).

### Elimination Half‐Lives

The fitting procedure permitted estimation of the elimination half‐lives and therefore the Gd content at 1 day after the last administration (Table 2). The extrapolated Gd concentrations in the cerebellum (1.062, 1.120, and 1.341 nmol Gd/g for gadoteridol, gadoterate and gadobutrol, respectively) and cerebrum (1.063, 1.042, and 1.033 nmol Gd/g, respectively) at 1 day after the end of dosing were statistically comparable for the three GBCAs with *P* values for cerebellum and cerebrum, respectively, of 0.73 and 0.92 (gadoteridol vs. gadoterate), 0.14 and 0.87 (gadoteridol vs. gadobutrol), and 0.06 and 0.94 (gadoterate vs. gadobutrol). Thereafter, more rapid elimination of Gd from both cerebellum and cerebrum occurred for animals given gadoteridol (Figure 2), reflecting the shorter half‐life of elimination for this GBCA. Conversely, in the skin, significantly lower levels of Gd were determined already at 1 day after the end of gadoteridol dosing compared with at the same timepoint after gadoterate or gadobutrol, despite the statistically similar half‐lives of elimination for the three agents (gadoteridol vs. gadoterate; *P* = 0.7; gadoteridol vs. gadobutrol: *P* = 0.76; gadoterate vs. gadobutrol: *P* = 0.93).

**TABLE 2:** Estimated Gd Content (± error) at 1 Day After the End of Dosing and Elimination Half‐lives (days), Calculated Assuming First‐Order Kinetics

	Estimated Gd Content at 1 Day After the End of Dosing (nmol Gd/g)	Half‐life (days)
Cerebellum		
Gadoteridol	1.062 ± 0.160	9.13 ± 1.60
Gadoterate	1.120 ± 0.055	15.91 ± 1.26***
Gadobutrol	1.341 ± 0.106	13.80 ± 1.61*
		
Cerebrum		
Gadoteridol	1.063 ± 0.163	8.64 ± 1.50
Gadoterate	1.042 ± 0.108	17.54 ± 3.09**
Gadobutrol	1.033 ± 0.074	17.76 ± 2.20***
		
Skin		
Gadoteridol	8.414 ± 2.797	3.92 ± 0.92
Gadoterate	24.879 ± 5.716**	4.39 ± 0.77
Gadobutrol	22.932 ± 5.555*	4.30 ± 0.78
		
Blood		
Gadoteridol	0.436 ± 0.021	9.47 ± 0.54
Gadoterate	0.225 ± 0.020***	11.28 ± 1.30
Gadobutrol	0.263 ± 0.005***	12.53 ± 0.32***

The significance of differences between groups is shown as *P ≤ 0.05, **P ≤ 0.01, ***P ≤ 0.001 vs. group 1.

The half‐life of Gd elimination from blood was significantly shorter in terms of days for gadoteridol compared to gadobutrol but not significantly different (*P* = 0.2) when compared to gadoterate (Table 2). The half‐lives of Gd elimination from blood for gadoterate and gadobutrol were not significantly different (*P* = 0.35). The extrapolated Gd levels at 1 day after the end of dosing, as well as measured Gd levels at all tested timepoints, were significantly higher for gadoteridol, further suggesting more rapid ongoing clearance from soft tissue organs, prior to renal elimination.

## Discussion

We compared Gd levels in rat cerebellum, cerebrum, skin, and blood at 1, 2, 3, and 5 weeks after repeated daily administration (4 consecutive days a week for 5 consecutive weeks at a daily dose of 0.6 mmol/kg, as utilized in previous studies^5^, ^12^, ^13^) of three macrocyclic GBCAs. Gd levels in cerebellum, cerebrum, and skin were significantly lower after gadoteridol administration than after gadoterate and gadobutrol at all tested timepoints. Conversely, significantly higher Gd levels were noted in blood after gadoteridol than after gadobutrol at 1, 2, and 3 weeks and after gadoterate at 1, 2, 3, and 5 weeks.

In a previous long‐term study of GBCA elimination in rats, Jost et al^14^ showed that the levels of Gd at 26 and 52 weeks after administration of a total cumulative dose of 14.4 mmol/kg (4 consecutive days a week for 2 consecutive weeks at a daily dose of 1.8 mmol/kg; i.e. higher than the human equivalent, clinically approved dose^15^) were similar, concluding that the elimination profiles of the macrocyclic GBCAs were essentially identical. However, the starting point for their study was at 5 weeks after the end of dosing, at which point the mean concentration of Gd determined in the cerebellum was significantly lower for gadoteridol (0.19 nmol/g) than for gadoterate (0.54 nmol/g) and gadobutrol (0.63 nmol/g). A recent joint initiative to harmonize methodological aspects for the nonclinical assessment of Gd retention suggested that a time interval up to 4 weeks after the last administration would be appropriate to assess short‐term elimination, since this is the period during which faster excretion of soluble Gd‐species occurs.^22^ Our study addresses the initial 5‐week period neglected by Jost et al,^14^ confirming not only their findings of lower Gd levels at 5 weeks for gadoteridol than for gadoterate and gadobutrol, but also, additionally, providing Gd clearance kinetics from cerebellum, cerebrum and skin within the 5‐week time period. In assessing four different timepoints after administration, our findings also confirm those of others who determined Gd levels in rat brain and other soft tissue organs at 7 days^11^ and 28 days^12^, ^13^ after GBCA administration.

Importantly, our study also addresses potential criticism of the fact that prior studies on Gd elimination in the first days and weeks after administration drew conclusions concerning Gd elimination kinetics based only on one post‐administration timepoint. Our findings confirm that significantly lower levels of Gd were present in rat cerebellum, cerebrum, and skin at all post‐administration timepoints after administration of gadoteridol compared with gadoterate and gadobutrol. Moreover, determination of Gd levels at four different timepoints allowed us to calculate decay curves for Gd concentration in the different tissues and to determine half‐lives of elimination for the different macrocyclic GBCAs over the initial 5 weeks after administration. This in turn allowed us to extrapolate Gd levels to just 1 day after the end of dosing. For the cerebellum and cerebrum, the half‐lives of elimination were considerably shorter for gadoteridol than for gadoterate and gadobutrol. Thus, more rapid elimination of Gd was seen from animals given gadoteridol beginning within approximately 1 day after the end of dosing. A different elimination profile was seen for the skin, where the extrapolated Gd level at day 1 was roughly three times lower for gadoteridol than for gadoterate and gadobutrol. This possibly reflects faster clearance of gadoteridol immediately after the GBCA administration phase. Given that skin is a collagen‐rich tissue, this finding is consistent with findings from a recent *in vitro* study, which showed that gadoteridol accumulates to a lower degree in collagen than both gadoterate and gadobutrol.^23^ Our findings for the skin are also consistent with those from a previous study of long‐term Gd retention in the skin of rats,^24^ which indicated considerably lower levels of retained Gd at early timepoints after a cumulative dose of 12.5 mmol/kg bodyweight (approximately 23 nmol Gd/g skin for gadoteridol at 3 days after the end of dosing compared to 50–60 nmol Gd/g skin for gadoterate and gadobutrol). Consistent with our findings, they showed that by day 35 after the end of dosing, the Gd levels in the skin were lower for gadoteridol (1 ± 1 nmol Gd/g), than for both gadoterate and gadobutrol (2 ± 1 nmol Gd/g).^24^

As noted elsewhere,^12^, ^13^, ^25^ reasons for the more rapid clearance of Gd from brain and soft tissue organs after gadoteridol administration can be ascribed to differences in the molecular features of this GBCA compared to the other macrocyclic GBCAs. In this regard, faster diffusion and clearance from brain interstitium and soft tissue organs would be favored for molecules that have low‐molecular weight and molecular features that minimize potential interaction with surrounding tissue matrix. Of the three macrocyclic GBCAs evaluated, gadoteridol has a low‐molecular weight of 558.7, which is similar to that of gadoterate (558.6) but lower than that of gadobutrol (604.7), and also the greatest lipophilicity as demonstrated by the butanol/water partition (P) coefficient (−1.98 log P_butanol/water_ vs. −2.87 and − 2.0 log P_butanol/water_, for gadoterate and gadobutrol respectively); both of these features favor its more rapid clearance.^12^ The greater lipophilicity of gadoteridol is particularly interesting given that the glymphatic system is considered a principal route of elimination of retained Gd^26^ and that it is known that this system transports lipophilic molecules more efficiently than hydrophilic molecules, thereby reducing their diffusion into the brain parenchyma.^27^ Concerning the lower potential of gadoteridol for interaction with surrounding tissue matrix, this reflects 1) the fact that the gadoteridol molecule is neutral and so would be expected to have fewer impediments to diffusion caused by ionic interactions than would the ionic gadoterate molecule, and 2) the gadoteridol molecule has only one hydroxy group and so would be expected to have fewer impediments to diffusion caused by hydrogen bonding than the gadobutrol molecule which has three hydroxy groups.^12^, ^13^ Although a recent study in rabbits revealed no statistically significant differences between gadoteridol and gadobutrol in terms of mean Gd concentrations in the cerebellum and cerebrum at 2, 6, and 12 weeks after administration, individual values were consistently slightly higher for gadobutrol at all timepoints.^28^ Unfortunately, the use of geometric means and geometric standard deviations differed from the arithmetic means and standard deviations used in this and other long‐term elimination studies,^29^ preventing direct comparison of findings.

Concerning Gd concentrations in the blood, significantly higher levels were observed for gadoteridol compared to gadobutrol at 1, 2, and 3 weeks after the last administration and compared to gadoterate at all tested timepoints, with marked differences noted during the first 2 weeks. Extrapolation also determined markedly higher Gd levels in blood at just 1 day after the end of dosing. Interestingly, nonsignificantly higher levels of Gd in plasma were also noted after gadoteridol compared to gadobutrol in a study involving rabbits.^28^ Although further studies are needed, it is likely the higher levels of Gd in the blood compartment in the first days and weeks after gadoteridol administration reflect greater ongoing clearance from brain, skin, and other soft tissue organs, prior to renal elimination.

### Limitations

A possible limitation of our study is that we did not look at Gd elimination beyond 5 weeks. This is because the principal difference in elimination appears to occur within the first days and weeks after administration. Moreover, Jost et al^14^ previously looked at long‐term elimination of Gd after exposure to macrocyclic GBCAs and noted no significant differences at 26 and 52 weeks. Given that 1 rat year corresponds to approximately 30 human years,^30^ the initial 5‐week period evaluated in our study would correspond to approximately three human years during which faster clearance of gadoteridol might be expected if findings in rats are suggestive of findings in humans. This would imply that less Gd is available for distribution and retention if gadoteridol were used. Interestingly, Jost et al^14^ reported that at the termination of their study (52 weeks) up to 90% (gadobutrol), 94% (gadoterate), and 81% (gadoteridol) of the Gd concentration present at 5 weeks had been eliminated. Given that the Gd concentration at 5 weeks was roughly three times lower for gadoteridol than for gadoterate and gadobutrol, this further indicates that a much larger proportion of Gd was eliminated in the first weeks after gadoteridol administration than was the case for gadoterate and gadobutrol.

Another possible limitation is that only male Sprague Dawley rats were used. Previous studies have used either male animals only^4^, ^11^, ^12^, ^13^, ^14^ or female animals only.^5^, ^29^ Although no effect of gender is expected for Gd retention, to our knowledge, no studies have yet compared male and female animals in terms of the levels of retained Gd. Further work is needed to determine whether gender‐related differences are apparent. Future work might also look at Gd retention and elimination from other tissues and organs. A recent study looked at Gd retention within multiple rat organs after intra‐articular GBCA administration and noted differences between the linear agent gadodiamide and the macrocyclic agent gadobutrol.^31^ It would be of interest to determine whether differences exist also among the macrocyclic GBCAs.

## Conclusion

Our study demonstrates that gadoteridol is eliminated more rapidly than gadoterate and gadobutrol in the first 5 weeks after administration. Gd levels in cerebellum, cerebrum, and skin were significantly lower after administration of gadoteridol at all timepoints investigated and decreased more rapidly over the course of the study. Gd levels obtained from extrapolation revealed that differences between the macrocyclic GBCAs were already present at 1 day after the end of dosing.
